# The Challenge of Measuring Exercise: Advancing Metrological Barriers in Wearable Sensing

**DOI:** 10.2196/79347

**Published:** 2025-12-10

**Authors:** Jennifer L Corso, Evan Peikon

**Affiliations:** 1 NNOXX Inc Seattle, WA United States

**Keywords:** photoplethysmography, PPG, muscle oxygen saturation, activity tracker, near-infrared spectroscopy, NIRS, wearable technology, remote patient monitoring, exercise

## Abstract

Regular physical activity offers extensive health benefits, yet current consumer wearables struggle to accurately quantify these effects at an individualized level. Sensor performance often falls short due to susceptibility to interferences, nonstandardized validation, and reliance on indirect estimations. Further, sensors often cannot capture or account for disparities in measurement types, populations, and physiological or anatomical characteristics, nor can they account for how different exercise modalities affect results on a personalized scale. There is a drive for developers to refine the impact of how we measure the benefits of exercise, improving the usefulness of data through advanced optical modeling and spectroscopic applications. This review critically examines the shortcomings of prevailing noninvasive measurements and techniques used in common, commercially available fitness trackers and describes why it is difficult to quantify the effects of exercise as an individualized, quality-based metric. Next, we discuss newer sensing applications that attempt to curtail known limitations, some of which may unveil novel biometric insights through differentiated approaches, bridging gaps not only in technological advancement but also in physiological metrology. In conclusion, we believe that new sensing techniques should explore solutions beyond population-based statistics and aim to provide an individualized understanding of a person’s response to exercise, while also reducing disparities in personalized health monitoring. The results could lead to a more effective understanding of exercise efficacy and its impact on performance management and clinical outcomes.

## Introduction

### Background

The phrase “sitting is the new smoking” has gained prominence as a compelling call to action, urging individuals to reconsider the implications of inactivity on their overall health and longevity. Among US adults, the obesity rate has exceeded 41% of the population according to the most recent reports provided by the Centers for Disease Control and Prevention, and heart disease, largely preventable via exercise, has been the leading cause of death since 1950 [1,2]. Despite the growing body of evidence highlighting the negative impacts of a sedentary lifestyle on lifelong health outcomes, many Americans still struggle to incorporate regular physical activity into their routines. Studies consistently show that engaging in regular exercise significantly boosts cardiovascular and musculoskeletal health, ultimately leading to reductions in all-cause mortality [3-5]. Further, large cohort studies indicate that participation in sporting activities may reduce all-cause mortality by nearly 40% [6], which further stresses the positive implications of fitness on overall longevity.

A 2023 meta-analysis published in the *British Journal of Sports Medicine* examined dose-response associations to physical activity in more than 30 million participants across 94 cohorts [7]. The study found that moderate physical activity significantly reduces chronic disease risk and suggested that even half of the recommended 150 minutes of weekly exercise could prevent 1 in 10 premature deaths and improve overall health outcomes. Exercise continually stands out as one of the most effective interventions for improving the quality of life [8], and the addition of activity trackers has been shown to improve exercise-related outcomes in both clinical and healthy populations [9]. The use of consumer wearables is linked to improved body composition and overall fitness through increased amounts of physical activity when compared to nonusers.

The cardiovascular benefits conferred by engaging in physical activity are robustly documented [10]. Regular exercise is linked to a lower risk of developing atherosclerotic heart disease and hypertension, largely due to enhanced vascular reactivity and an increased expression of endothelial nitric oxide synthase (eNOS) [11]. Enhanced eNOS activity boosts overall nitric oxide synthesis, contributing to lower vascular resistance and reduced arterial blood pressure, consequently mitigating risks associated with all-cause mortality [12].

The hematological benefits of exercise are equally significant and dose dependent. Regular physical activity leads to increased plasma volume, erythrocyte mass, and erythropoietin synthesis, which are beneficial for endurance performance and lead to improved aerobic capacity [13]. The current gold standard for assessing aerobic capacity and overall cardiovascular fitness is through the measurement of an individual’s maximum volume of oxygen consumed (VO_2max_) using a graded exercise test and gas analysis. A higher VO_2max_ indicates a greater physiological capacity to uptake and use oxygen during aerobic exercise and correlates to a lower risk of cardiovascular disease.

It is abundantly clear that the acute and chronic health benefits of exercise are research supported and plenty. Physical activity significantly influences tissue perfusion, oxygen delivery, and consumption, as well as muscular strength, cognitive function, and overall longevity [14,15]. However, quantifying the benefits on a personalized scale poses substantial challenges outside of controlled laboratory settings.

### Review Objectives

Currently, there is no standardized, user-friendly method to assess the *benefit* of exercise that is tailored to individual physiological dynamics. This creates a gap in the practical understanding of the effective impact of physical activity on an individual’s health, as the biometrics used today are also limited. Therefore, we cannot easily answer the following question: What is the *efficacy* of exercise for an individual? At present, the general public is unable to quantify how well they acutely respond or adapt, thereby limiting any actionable modifications to maximize both acute and chronic exercise quality, objectively.

The aim of this viewpoint is to provide an overview of the current state and limitations of exercise metrology, followed by potential solutions to measuring exercise efficacy on an individualized scale. First, we review the limitations of current optical sensors, applications, and associated physiological biometrics commonly used in the noninvasive measurement of exercise performance. Next, we discuss various technological developments that attempt to unveil new information in relation to measuring exercise efficacy, with the ultimate goal of generating personalized exercise physiology data to improve biometric insights. We then conclude with how we believe the measurement science should move forward.

## Measurement Challenges in Exercise and Health

Emerging technologies, particularly wearable devices and artificial intelligence/machine learning (AI/ML)–powered algorithms, are beginning to bridge these gaps by enhancing access to one’s personalized health data. Integrated platforms deliver insights into disease risk and behavioral outcomes related to physical activity, albeit within certain limitations, such as data quality and interoperability [16,17]. For elite athletes, even marginal improvements in the quality of exercise data can have substantial effects on performance outcomes. However, technological enhancements can sometimes teeter on ethical lines: Do the data provided simply enhance training improvement or push natural adaptations outside the bounds of the current understanding of sport and medicine [18]? Can technologies do this accurately and lend more confidence to outcomes, or do the data raise more questions and create controversy? How are we contextualizing the information based on the known performance attributes of current sensors? For clinical patients, the current generation of noninvasive devices fails to meet the stringent reliability standards required to improve health outcomes, thereby making medical therapeutics a market relatively void of effective user-friendly wearables [19,20].

### Limitations of Photoplethysmography in Biometric Assessment

**Figure 1:**
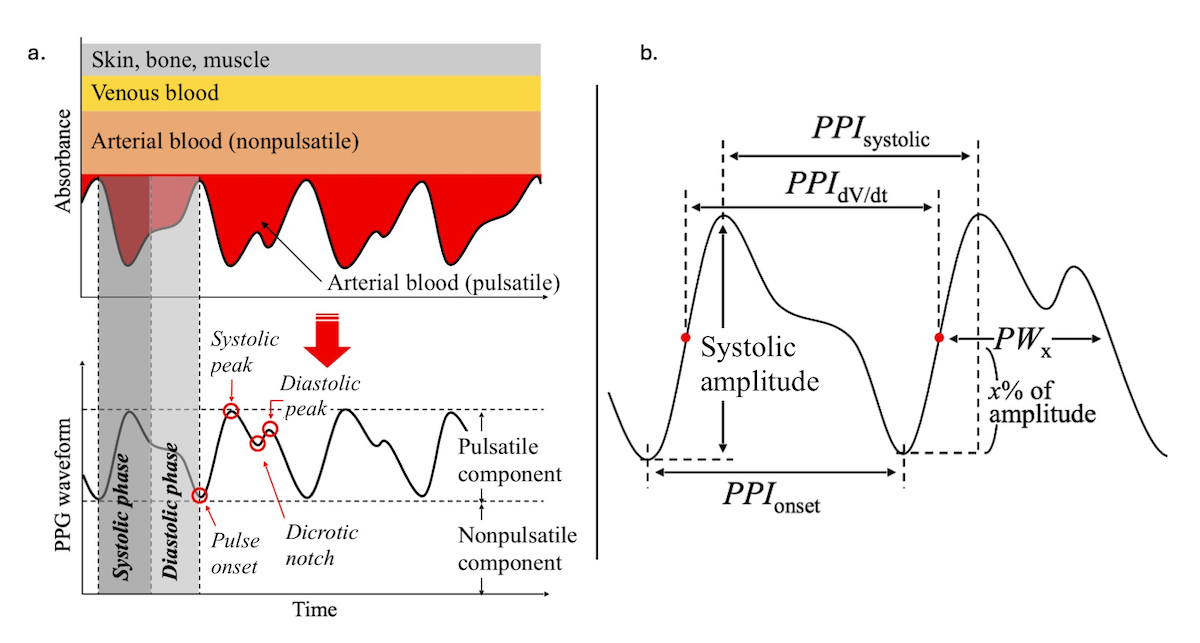
(a) Principle of using PPG and waveform features used to infer physiological parameters. (b) Systolic and diastolic phases. PPG: photoplethysmography; PPI: pulse-pulse interval; PPIdV/dt: maximum dV/dt of adjacent pulse-pulse interval; PPIonset: adjacent pulse-pulse onset interval; PPIsystolic: adjacent pulse-pulse interval; PWx: pulse width at x% of the systolic amplitude. Reproduced with permission [22].

Despite advancements in wearable technologies, significant limitations exist when using such devices for assessing the benefits of exercise for specific applications, such as for professional athletics and aging adults. Many devices use light-emitting diode (LED) photodiodes at specific wavelengths coupled with photoplethysmography (PPG) to estimate health metrics, such as pulse rate (eg, heart rate [HR]). Attempts have been made to expand the use of the PPG signal to monitor more complex variables, such as cardiac output (CO) and blood pressure [21]. Figure 1 illustrates the general principle of using PPG to assess biometric variables by analyzing a variety of waveform features [22].

PPG, which optically measures changes in blood volume over time, is susceptible to a variety of interferences. PPG primarily reflects changes in peripheral blood volume, which is influenced by factors such as temperature, sympathetic nervous system activity, and certain medications. These factors can alter peripheral vascular tone, affecting the PPG waveform and potentially leading to inaccuracies in biometric estimations. Further, the optical properties of living tissue vary by tissue type, blood and water content in the tissue, collagen, melanin, and any connective tissue fiber development [23]. It is widely known that biases in the data stem from interferences, such as melanin, and the US Food and Drug Administration (FDA) has proposed new guidance to reduce performance discrepancies specifically in pulse oximetry [24,25].

**Figure 2:**
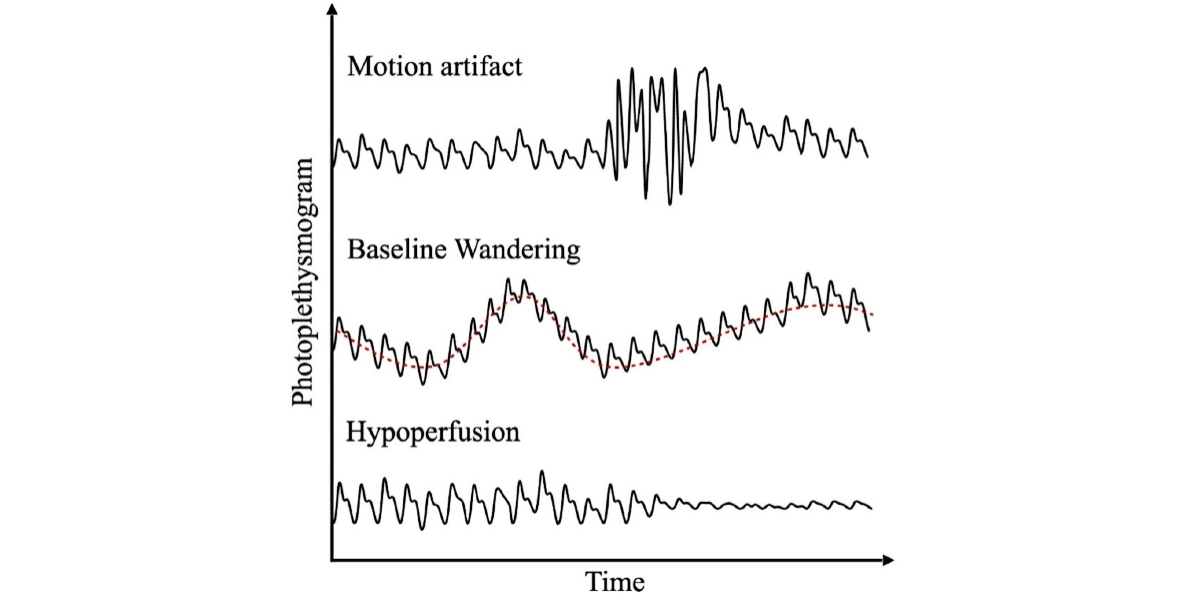
PPG signal distortion due to motion artifact, baseline wandering, and hypoperfusion. PPG: photoplethysmography. Reproduced with permission [22].

Figure 2 provides three examples illustrating how the PPG signal may distort over time [22]. Generally, this happens when interferences, such as those factors explained previously, influence the signal and degrade the ability to interpret the targeted signal features clearly. Motion artifacts and signal interference are paramount and can compromise the signal-to-noise ratio of the PPG measurement, thereby limiting physiological insights [26]. Signal corruption stemming from motion can be estimated and rectified using applications such as quadrature reference signals or other correlational filters [27]. Other interferences include baseline drift, baseline wandering, and stochastic noise. Further, body location, skin-to-sensor pressure at the measurement location and breathing characteristics also significantly impact waveform characteristics, including the mean amplitude, dicrotic notch time, and reflection index [28]. Because quality and reliability are crucial features of data applicability, variability among sensors makes it difficult to measure these attributes and to ascertain their impact on outcomes. A lack of stringent validation standards for PPG-based devices strongly limits their analytical utility and reliability.

These limitations may necessitate the use of auxiliary hardware for more accurate data collection. This is the case in arrhythmia detection, which has propelled itself into modern consumer wearables. Additionally, PPG cannot adequately capture certain vascular dynamics, such as reflective effects on the vessel expansion interrogated after transmission of the waveform, making estimates for parameters such as blood pressure challenging and leading to low application validation in clinical populations [29,30].

In general, device limitations impact the usability of a variety of commercially available and common health metrics. The data are only as useful as the metrological capabilities.

### Step Counts and Health Outcomes

The recommendation of taking 10,000 steps per day originated from the Japanese company Yamasa, which launched a pedometer called *Manpo-Kei* around 1965, shortly after the Tokyo Olympics. The pedometer name translates to “10,000 steps meter.” The campaign promoted this number with the catchy slogan “Let’s walk 10,000 steps a day,” which coincided with the rise of walking clubs in Japan at that time. The intention was to encourage people to be more active.

The current recommendation of 10,000 steps per day set forth by the American Heart Association (AHA) was built upon the original Japanese campaign. The aim of the AHA was to promote activity using an accessible benchmark, with a goal to reduce chronic disease risk. The step count quantity has been challenged by recent research. Though not an exhaustive list, several recent studies indicate that lower step counts may still be effective for improving cardiovascular and overall health, thereby prompting a re-evaluation of the necessary number of steps for optimal health outcomes and improvements in cardiovascular fitness [4,31-34]. A range of 8000-9000 steps per day may protect against diseases such as hypertension, diabetes, sleep apnea, major depressive disorder, and obesity [32]. Even lower counts, roughly 4000 steps, may lower mortality rates significantly [4,33].

Steps are considered objective, assuming the device measuring them has a relatively low margin of error and the exerciser is using the device as indicated by the manufacturer. If these attributes are not maintained, steps can be an inaccurate depiction of exercise quantification, as gait is often misinterpreted by electronic or electromechanical pedometers and inertial measurement units (IMUs)—accelerometers and gyroscopes used to measure the body’s angular rate, force, and orientation. Over- or underestimating a true value is a significant limitation, with errors sometimes surpassing 10%, depending on body placement and validation criteria [35,36].

Often, if an individual is walking with a device located on the wrist and the arm is static, for example, while pushing a stroller or shopping cart, the pedometer does not register some if not all of the steps accrued. This is due to low or no cyclic motion registered by the device sensor. Further, metrological inconsistencies may conflict with Global Positioning System (GPS)–derived data. For example, if 2000 steps are roughly equivalent to 1 mile when using an average stride length, then an over- or underestimation may show a user traveled farther or less via GPS, which may be confusing.

The intensity of physical activity associated with a specific step count is often inadequately assessed by many wearable devices unless additional parameters, such as HR, are integrated. Standard step-counting metrics can fail to capture the differences in activity intensity; for instance, a person may accumulate the same number of steps through both leisurely walking and brisk walking, yet the cardiovascular and metabolic benefits vary significantly [35].

Furthermore, vigorous activities of daily living (ADLs), such as lifting heavy objects or engaging in high-intensity interval training, are typically not reflected in step counts. These forms of activity can yield significant health benefits, particularly for populations such as older adults or individuals with chronic conditions [32,37]. Other types of activity are necessary for people with disabilities that limit their ability to walk, such as strength training.

### Heart Rate as a Proxy for Exercise Intensity

HR is frequently used as a proxy for gauging exercise intensity and is typically assessed via PPG in consumer wearables. However, this methodology is not without inherent limitations: PPG’s reliance on pulse wave analysis is vulnerable to motion artifacts, variations in the quality of skin contact, and signal loss during irregular movement, all which can severely compromise measurement accuracy [38]. Improvements in validation testing criteria, denoising, and waveform peak identification are being enhanced with the incorporation of trained deep neural networks (DNNs) [39,40].

Delays in adaptive algorithms limit the accuracy of HR to nonambulatory conditions and, to a lesser extent, during steady-state exercise. Many algorithms use sliding windows (eg, 8- or 12-second processing windows) to estimate HR across a predesignated timespan. This approach introduces lag, preventing real-time detection and tracking of rapid HR changes, such as during interval training [41]. The error increases dramatically, and data are often unusable in these instances; in addition, the last reportable value is carried forward until the error decreases below a predetermined threshold of acceptable accuracy. In general, HR estimates are only reliable during rest or in steady-state conditions. However, inaccuracies may still be present during sleep, when HR may drop below 50 beats per minute in trained adults or those with underlying conditions.

Lastly, HR is not a direct measure of the benefits of exercise in all cases, such as during strength training. The estimate is not equally validated across all forms of exercise or in contraindicated groups [42]. Additionally, users often lack the requisite knowledge to interpret maximum HR and its implications for training regimens, thereby leading to potential errors in exercise intensity assessments [43].

### VO_2_ and VO_2max_ Measurements

Simply, the volume of oxygen consumed (VO_2_) is the volumetric difference between oxygen inspired and oxygen expired over a measured amount of time (1 minute). VO_2max_ is the maximum amount of oxygen one’s body can consume at absolute maximal exertion. VO_2max_ is used as a gauge to indicate an individual’s overall cardiovascular fitness. Commercially available wearables often rely on HR data to estimate VO_2_ and VO_2max_, but this approach can yield significant discrepancies from actual values, with variations reaching as high as 20% [44]. The estimate is only as good as the HR metric, which is estimated from a pulsatile waveform.

Accurate assessment of VO_2max_ typically necessitates maximal exertion testing conducted under laboratory conditions, which is rarely achievable in nonclinical settings. For accurate estimation, VO_2max_ testing must take participants to volitional fatigue, validated by physiological markers, such as blood lactate concentration, respiratory exchange ratio (RER), and individual rate of perceived exertion (RPE) [45]. Most individuals deriving a VO_2max_ estimate from commercial wearables do not achieve a true value because of the absence of rigorous testing protocols. In general, users may reach a “peak” value (VO_2_peak), at best.

A user must know their maximum HR and resting HR. There are several formulas that can be used to calculate maximum HR, some more relative to an individual than others and often relying on an age to predict the value. Other methods use a heart rate ratio method (HRRM) in tandem with maximum HR. Because maximum HR is an estimate and linearly related to age, simple formulas may not account for the cardiorespiratory fitness of an individual [46,47]. Differing VO_2max_ values may be acquired for a given person, depending on the device being used, the test being given, and the user experience level, coupled with the different maximum HR equations. Further, many wearables overestimate the resting HR, particularly for endurance athletes, and they may even be outside the validated algorithm range, with many devices having no validation below 40-45 beats per minute (bpm). Moreover, factors such as dehydration and ambient temperature can further distort HR-VO_2_ relationships, further complicating accuracy [48,49].

### Metabolic Equivalents and Energy Expenditure

Metabolic equivalents (METs) serve as a useful framework for quantifying the energy expenditure (EE) of a given physical activity. One (1) MET is defined as a VO_2_ rate of 3.5 mL/kg/minute, equal to an adult’s resting metabolic rate [45].

METs are used as a relative way to compare the EE of a given activity to someone’s resting metabolic rate over a given unit of time. Activities with higher MET values can be used to assign a specific intensity of exercise, help personal trainers and practitioners plan an exercise routine, and also estimate the number of calories burned during exercise.

For instance, brisk walking registers at approximately 3.5-4.0 METs, while running at 7 mph is around 7.5 METs [50]. However, discrepancies arise when using standard MET values for individuals of varying body weights, as these calculations are based on an average body weight of 70 kg (154 lb). Consequently, comparisons across individuals with different body compositions and adiposity may yield inaccurate assessments [51].

Further, not all METs are equal across sport or exercise type, particularly if full body movement is not involved in the activity [52]. Often, occupational activities and ADLs that elevate HR are not counted in the overall time spent at a given MET.

Moreover, the public’s understanding of METs is often restricted to clinical or academic contexts, complicating their practical application. The complexity of these metrics can deter individuals from leveraging them effectively in their personal fitness regimens [53]. Generally, people don’t want to perform math to understand how many kilocalories are burned at a given VO_2_ and MET value. With accessibility of simpler metrics, they often default to using HR to understand how hard they are working.

### Cardiac Output Measurement

The CO (Q) reflects the heart’s ability to meet metabolic demands, providing insight into cardiovascular function. It is particularly useful in clinical contexts such as heart failure management [54]. It is calculated by multiplying HR by stroke volume and reflects the volume of blood ejected by the heart per minute. It can be estimated using the Fick principle during exercise testing. It is typically measured using thermodilution, which is invasive and requires an arterial catheter, and can also be estimated with other methods, such as echocardiography and esophageal Doppler.

Recent advancements in technology have enabled the development of devices that purport to measure the CO using PPG, with the signal most often acquired from the fingertip. PPG and electrocardiographs (ECGs) have been used in tandem to aid in determining stroke volume by measuring pulse transit times from the ECG’s pulse rate and the pulse measurement estimated by the PPG device. Stroke volume is estimated as a function of the slope transit time and by analyzing the pulse contour of the primary peak in the waveform, though the accuracy of this estimation can be influenced by changes in vascular tone and afterload [55,56]. Limitations exist around PPG’s accuracy in determining stroke volume alone [57] and also inferring physiological measurements, such as total peripheral resistance. Some of the PPG applications exhibit error margins approaching 40% when compared to thermodilution and echo Doppler, including those in use cases where patients are surgical in nature [58-61]. Current developments use robust ML methodologies in an attempt to satisfy performance accuracy closer to thermodilution in assessing hemodynamics [62]. Please see Table 1 for an overview of the previously discussed measurements.

**Table 1:** Summary of noninvasive physiological measurement methods and limitations used widely in the noninvasive assessment of exercise performance.

Measurement	Method	Validation reference standard	Measurement limitations
Steps	Accelerometry (eg, IMU^a^)	ActiGraph; manual step counting with video recording	Unable to account for intensity or activities not reflected by steps (eg, resistance training) [32,35,36] Misinterpretation of gait by accelerometer or misalignment with GPSb-derived data [63] Error dependent on anatomical location (1% to >10%) [35,36] More or less beneficial based on risk profile of individual [4,32,33]
HR^c^	PPG^d^ (eg, waveform analysis, peak detection, calculation of bpm^e^)	ECG^f^, chest strap HR monitor	Susceptible to motion artifact limiting accuracy during motion [38] Susceptible to sliding windows limiting accuracy during intensity changes [41] Not validated or equal across all forms of exercise or cardiac rhythms [42]
VO_2_^g^, VO_2max_^h^	Typically inferred from HR using PPG	Laboratory metabolic testing using gas analysis	Limited by accuracy and calculation of HR and maximum HR, as well as factors that affect HR [44-47,49]
MET^i^ and EE^j^	Derived from VO_2_ (typically inferred from HR if delivered by a PPG-based wearable)	1 MET, which is a VO_2_ of 3.5 mL/kg/minute standardized to 70 kg body mass	Inequality of MET values across different body compositions [51] Inequality of MET values across exercise types if full body movement is not involved or during ADLsk [52] Not well understood by all users/prone to misapplication [53]
CO^l^ (Q)	PPG (eg, estimation of stroke volume and HR) with or without other methods (eg, ECG, impedance cardiography)	Transpulmonary thermodilution, transthoracic or esophageal echo Doppler	High error potential due to physiologic influences (eg, afterload, total peripheral resistance, vascular tone) [55,58-62] Limitations in interpretation of stroke volume, which may require additional/coordinated inputs (eg, PPG+ECG) [55,57,64]

^a^IMU: inertial measurement unit.

^b^GPS: Global Positioning System.

^c^HR: heart rate.

^d^PPG: photoplethysmography.

^e^bpm: beats per minute.

^f^ECG: electrocardiograph.

^g^VO_2_: volume of oxygen consumed.

^h^VO_2max_: maximum volume of oxygen consumed.

^i^MET: metabolic equivalent.

^j^EE: energy expenditure.

^k^DL: activity of daily living.

^l^CO: cardiac output.

## Overcoming Barriers to Effectively Measure Exercise

To make general strides in measuring exercise, two interconnected points need to be addressed, as new technology ideally gleans new biometric insights:

- Some biometric limitations exist because of technological methods. What additional sensing capabilities are available to help improve current measurements and improve the overall measurement of exercise efficacy?
- From newer sensing technologies, what additional metrics can be derived that may lead to a better understanding of an individual’s physiology, therefore lending an improved representation of exercise effectiveness, efficiency, and overall efficacy of application?

### Advancements in Sensing Capabilities

Noninvasive spectroscopic methods are still fairly limited to the interrogation of the dermis and the relevant substructures and chemistries contained within. Several microneedle studies analyzing dermal blister fluid have refined the understanding of what is quantitatively represented in the skin’s interstitial fluid, both somatically (eg, from the blood) and locally (eg, produced by regional cells) [65]. Measuring these putative biomarkers noninvasively poses unique hurdles, as concentrations are typically low and therefore difficult to quantify with acceptable accuracy and precision [66]. The challenge can be exemplified by the multidecade pursuit to measure glucose concentrations in the skin without relying on minimally invasive techniques [67].

Sensor capabilities are expanding, from material selection to hybrid hardware and software integration. Newer optical approaches may hold promise for measuring individualized biomarkers and chemistries and provide continuous data streaming, some in real time, with little to no significant lag. Further, combining technologies may offer a way to obtain improved sensing performance compared to stand-alone applications. Novel methods, such as those incorporating concepts from link budgeting in telecommunications with bio-optics and AI, may produce sensors less prone to signal interferences and motion artifacts, allowing for cleaner and more accurate data collection during movement [68]. Advances in the engineering and use of novel materials and structures, including metamaterials, may enhance spectroscopic methods by confining light to subwavelength scales, improving integration and sensitivity across a broad optical spectrum [69].

Major advancements in PPG applications focus on dynamic, reconfigurable sensors to optimize signal quality using deep learning algorithms and neural networks [70]. Deep learning algorithms, such as convolutional neural networks (CNNs) and recurrent neural networks (RNNs), may improve the analytical performance of PPG-based devices by enhancing analysis of the shape and other characteristics of the waveform. Such algorithms can learn complex patterns and may access biometrics that would be immensely helpful in clinical scenarios [71,72].

Imaging photoplethysmography (iPPG) and remote photoplethysmography (rPPG) methods aim to provide biometric analysis using a contactless approach [73,74]. Off-body cameras are used to assess cardiovascular-based indicators via video by detecting fluctuations in skin blood volume between diastole and systole. Skin color analysis is performed, often of the face. In addition to deep learning and CNNs, spatial-spectral-temporal fusion (hybrid red-green-blue [RGB] camera and near-infrared [NIR] facial video) and spatial-temporal attention networks are a few methods that are applied to increase the accuracy biometric interpolation of video recordings [75-78].

Hybrid sensing is becoming increasingly popular, where PPG is also combined and packaged with additional hardware, such as electrical bioimpedance (BIA). Samsung’s new BioActive sensor is an example of a PPG-based noninvasive wearable that has not only expanded the number and type of LEDs but also cointegrated ECG and BIA in an attempt to improve metrological performance from data collected on the dorsal wrist [79]. The cointegration is not novel; however, the sensor includes blue, yellow, violet, and ultraviolet wavelengths, alongside an increase in green, red, and infrared (IR) LEDs, with a claim to improve performance compared to previous versions, in some instances nearly 30%. Other PPG applications related to sport science include interpretation of heat stress and overexertion through the monitoring of HR and heart rate variability (HRV), though the accuracy of these applications can be improved, with errors upward of 0.5°C [80].

Wearable microelectromechanical systems (MEMS) have been playing an increasing role in fitness-based sensing because of their ability to capture IR radiation emitted from the body. Broadband thermal MEMS are a major contributor in noninvasive thermal imaging and thermography, being able to detect fairly small changes in surface body temperature [81,82]. Generally, the sensors contain thermopile elements or microbolometers that operate across a broad IR spectrum, typically in the long-wave infrared (LWIR) range of approximately 8-14 µm. Thermopiles generate a voltage output proportional to the incident IR radiation, while microbolometers exhibit a change in electrical resistance as a function of absorbed thermal energy. When fabricated as focal plane arrays (FPAs), these MEMS-based detectors provide significantly higher spatial resolution compared to single-point thermal sensors, enabling detailed thermal mapping of complex surfaces [83]. Data translate to several performance-related use cases, such as estimating EE and body heat mapping, capturing information related to the physiological processes of both skeletal muscle heat creation and thermoregulation [81]. These advances may lead to potential improvements in understanding training load through entropy analysis, as well as recovery and fatigue assessment [84-86].

A significant amount of research and development has been focused on improving the bandwidth, energy efficiency, and scalability of biosensors. Advancements in short-wave infrared (SWIR) spectroscopy allows for the detection of absorption peaks from 900 to 2500 nm (depending on the source of reference), opening up a plethora of new technological opportunities in biometric monitoring [87-90]. The SWIR region contains absorption peaks for O-H bonds (1430 and 1940 nm), lipid-associated C-H bonds (1210, 1730, and 1760 nm), and collagen (1200 and 1500 nm) [88]. Through an expanded wavelength region, SWIR sensors can target new metrics and enhance the performance of current biomarkers, including hydration, body (dermal) temperature, albumin, glucose, lactate, ethanol, and others [90-92]. Several large players have expanded their R&D in silicon photonic biosensors, including Apple, building off many of the advancements previously made by technology pioneers specializing in the development of laser-based SWIR spectroscopy and photonics-based health sensors, including Rockley Photonics [93,94]. Depending on the overall optical solution, combining silicon photonics with microelectronics improves overall sensing capabilities, with an improved signal-to-noise ratio, lower propagation loss, a smaller overall package size, greater power handling, and overall enhanced performance. Combining SWIR diffuse reflectance spectroscopy with LED-based PPG may further refine and expand biomarker monitoring.

Unlike applications that target specific absorption peaks, applications using broadband-light spectroscopy (BLS) and white-light spectroscopy emit a wide spectrum of light to create a molecular “fingerprint” with relatively high specificity and sensitivity [95]. A label-free approach allows for the use of broadband light without tagged fluro- or chromophores to collect biometrics in a wearable form factor [96]. Some applications are combined with Raman-based techniques to reveal new bands in a given molecular fingerprint region [97]. Others use differentiated InGaAs photodetectors to obtain a broader range of light and a greater signal-to-noise ratio compared to silicon-based or traditional InGaAs solutions [98,99]. Broadband applications may use LEDs or laser diodes and be combined with additional noninvasive methods, such as laser Doppler and continuous-wave near-infrared spectroscopy (CW-NIRS) [100]. Further, emerging research indicates that boron nitride (BN) nanosheet-based photodetectors may offer ultrabroadband sensitivity—potentially spanning from deep ultraviolet to midinfrared—along with high thermal stability, spectral sensitivity, and self-powering capabilities, making them candidates for future applications in breath analysis and glucose sensing [101].

The application of deep optics using CW-NIRS may further improve wearable applications by defining specificity of tissue type through the use of multiple path lengths and algorithmically solving for absorption and scattering coefficients, which accounts for the heterogeneity of layered tissue from superficial to deep [68,102]. This allows the assessment of hematological variables with reliable performance when compared to invasive methods or common benchtop options, including frequency domain near-infrared spectroscopy (FD-NIRS) [103,104]. Unlike FD-NIRS, the CW-NIRS approach eliminates the need for modulated light sources and phase-sensitive detection systems, while maintaining improved signal quality. Recent recommendations aim to improve metrological best practices (data processing and interpretation) if NIRS is used to monitor cerebral and muscle oxygenation during exercise, particularly to account for noise generated by extracerebral tissue layers [105].

Albeit a snapshot of wearable sensing applications, all of these optical approaches are enhanced by the integration of advances in software and computing methods, complex large language models (LLMs), sophisticated and DNNs, and other ML methods including AI, which help further improve on-device processing and compensate for source and sensor limitations. Please see Table 2 for an overview of the previously discussed sensing advancements in exercise metrology.

**Table 2:** Summary of developing noninvasive sensing methods and emerging biometrics related to exercise physiology.

Sensing method	Sensing advancements	Emerging biometrics
PPG^a^	Hybrid sensing [77,79] Expanded wavelengths/photodiode options [79] MLb and computational advancements (eg, LLMsc, DNNsd, spatial-temporal applications) [70-72,75-78] Contactless methods (rPPGe, iPPGf) [73,74]	Heat stress/overexertion [80] Remote analysis of cardiovascular and respiratory vitals (eg, HRg, respiratoryrate) [73,74] Apnea, hypopnea [74]
Broadband MEMS^h^ thermography	High spatial resolution and improved thermal mapping [83]	Entropy analysis, EEi, body heat mapping/thermoregulatory analysis, training load analysis, recovery analysis [81,84-86]
SWIR^j^ spectroscopy	Expanded absorption peak range (900-2500 nm) [88-90] Improved signal-to-noise ratio, power handling, package size (chip) [94]	SWIR (diffuse reflectance spectroscopy): ethanol, hydration, dermal temperature, glucose, lactate, lipids [89-92]
BLS^k^/white-light spectroscopy	Label-free application [96] Improved InGaAs photodetector solutions [98,99] Ultrabroadband sensitivity, improved thermal stability with nanosheet photodetectors [101] Hybrid sensing [97,100]	Multi-omic and molecular fingerprinting, hydration, breath analysis, glucose [97,98,101]
FD-NIRS^l^ and CW-NIRS^m^	Enhanced signal quality and tissue specificity [104,106] Deep-tissue analysis [68,102,107]	Local hematological and oxygenation metrics (eg, hemoglobin, SmO2o), metabolic oxygen kinetics, cerebral oxygenation, bioactive nitric oxide (S-nitrosothiols), blood flow, injury analysis, recovery [102-104,107-111]

^a^PPG: photoplethysmography.

^b^ML: machine learning.

^c^LLM: large language model.

^d^DNN: deep neural network.

^e^rPPG: remote photoplethysmography.

^f^iPPG: imaging photoplethysmography.

^g^HR: heart rate.

^h^MEMS: electromechanical systems.

^i^EE: energy expenditure.

^j^SWIR: short-wave infrared.

^k^BLS: broadband-light spectroscopy.

^l^FD-NIRS: frequency domain near-infrared spectroscopy.

^m^CW-NIRS: continuous-wave near-infrared spectroscopy.

^o^SmO_2_: muscle oxygen saturation.

### Expanding Physiological Insights

Newer wearables, software applications, and ML tools are generating a variety of indirect metrics to better reflect human performance and fitness status. Many still use PPG-derived measurements with advanced software applications to obtain estimates of aerobic and anaerobic thresholds, the lactate threshold, VLa_max_ (lactate maximum), critical power, critical speed, and more. Although these metrics can provide valuable insights, most commercially available devices do not measure anything truly new and simply deliver an inferred and estimated biometric (eg, strain, recovery, or stress, derived from already-measured information, such as HR or HRV, or a combination that is undisclosed). The degree of accuracy of these inferences relies directly on both device placement and the performance of the underlying inputs, which are sometimes trade secrets. Overall, these metrics do not represent the *quality* or *significance* of exercise.

It is difficult to quantify how effective or efficient an acute session of exercise is for an individual. Often, population statistics are applied in an attempt to assess individualized outcomes relevant to performance or health, without attributing the nuanced limitations of either the technology (ie, an optical model or computational factors) or the body (ie, genetic limitations on oxidative capacity) or even disease. How does one know whether a type or intensity/duration of exercise is working in the short term? How does chronic training impact quality or efficiency? Body composition, improved strength, or decreases in the resting HR are routinely used to assess improvements (or the benefits of exercise) [45,112]. These attributes take weeks or months to denote relevant changes in physiology. It is not easily possible to assess the benefits of exercise during a workout, nor is it possible to assess recovery at a local level during acute periods between workouts.

Given the limited ability of current wearables to leverage individualized biostatistical data, a key question arises: *Which* biometrics paired with *which* sensing technology, either singlehandedly or in composite, yields the best indicators of exercise quality and efficacy? A holy grail in cardiovascular sports medicine is to measure the impact of activity on blood flow, not only to working muscle, but also to the myocardium, cerebral tissue, and nonworking tissues [113]. These data would provide a link between systemic and local metabolism, including oxygen kinetics, fuel homeostasis, and deviations during non-steady-state activity [114]. Further, blood flow information may provide unprecedented insights into vascular health, athletic injury, and recovery. Advancements in ultrasound techniques, such as superb microvascular imaging and laser Doppler, have opened a window to measuring tissue perfusion and directional blood flow at the 100-200 µm level in lesions, organs, and skeletal muscle [100,115-117] but are not field-practical and cannot be used easily during activity.

Insights gleaned from newly attainable, hematologically derived variables may allow for the measurement of exercise more effectively, and several NIRS techniques are making this realistic [102,104,107-109,118,119]. Obtaining local muscle oxygen saturation (SmO_2_), oxygen kinetics and oxidative metabolic activity, bioactive nitric oxide (*S*-nitrosothiols), local blood flow and perfusion, and other hemoglobin or flow-derived indicators during activity would be a monumental feat in cardiovascular assessment. Research supports the enhancement of key performance indicators with such biometrics, including equivalent or improved evaluation of lactate and ventilatory thresholds, as well as critical power in multiple sports [110,111,120,121]. Combined with information from hybrid-sensors, such as hydration and pulse rate, these data may be powerful, with substantial outcomes in denoting increments of value in both professional athletics and clinical care [20].

## Conclusion

The array of insights offered by modern wearables holds promise for understanding individual physical limitations, yet it often falls short in providing accurate and meaningful assessments of how well exercise is doing for someone. As technology continues to evolve, a more nuanced understanding of the relationship between exercise and health made possible through improved metrological techniques will be essential for fostering better lifestyle outcomes among individuals.

From the sensing advancements discussed previously, emerging measurements are being unveiled with physiological legitimacy and utility, including networked biomarkers, new relationships between biomarkers, and cause-and-effect chains between biomarkers. Progressing beyond PPG, developers can begin to offer alternative solutions to navigating improvements in physiological insights. With enough reliable data, we may begin to comprehend how measurable concepts, such as metabolomic fingerprinting and biological aging, may indicate where exercise may holistically slow the progression of disease and impact cellular senescence. Though this does not enhance individualized measurement of physiological performance, it does lend to long-term understanding of the influence of exercise on physiology from a real-time, continuous output.

The ultimate goal is to understand how individualized information related to exercise quality and efficacy may help patients, athletes, and practitioners reach therapeutic and training goals without relying on misapplied population-based statistics. Future research should focus on developing methods that bridge the gap between laboratory findings and real-world applicability through the use of real-time, wearable monitoring, which will ultimately empower users to make informed health choices based on truly personalized data.

## Abbreviations

ADL: activity of daily living
AHA: American Heart Association
AI: artificial intelligence
BIA: bioimpedance
BLS: broadband-light spectroscopy
bpm: beats per minute
CNN: convolutional neural network
CO: cardiac output
CW-NIRS: continuous-wave near-infrared spectroscopy
DNN: deep neural network
ECG: electrocardiograph
EE: energy expenditure
eNOS: endothelial nitric oxide synthase
FD-NIRS: frequency domain near-infrared spectroscopy
GPS: Global Positioning System
HR: heart rate
HRV: heart rate variability
IMU: inertial measurement unit
iPPG: imaging photoplethysmography
IR: infrared
LED: light-emitting diode
LLM: large language model
MEMS: electromechanical systems
MET: metabolic equivalent
ML: machine learning
NIR: near infrared
NIRS: near-infrared spectroscopy
PPG: photoplethysmography
PPI: pulse-pulse interval
rPPG: remote photoplethysmography
SmO2: muscle oxygen saturation
SWIR: short-wave infrared
VO_2_: volume of oxygen consumed
VO_2max_: maximum volume of oxygen consumed

## Data Availability

No new data were created or analyzed in this study. Data sharing is not applicable to this paper.
